# Risk-Return Relationship in a Complex Adaptive System

**DOI:** 10.1371/journal.pone.0033588

**Published:** 2012-03-30

**Authors:** Kunyu Song, Kenan An, Guang Yang, Jiping Huang

**Affiliations:** Department of Physics, State Key Laboratory of Surface Physics, and Key Laboratory of Micro and Nano Photonic Structures (Ministry of Education), Fudan University, Shanghai, China; National Research & Technology Council, Argentina

## Abstract

For survival and development, autonomous agents in complex adaptive systems involving the human society must compete against or collaborate with others for sharing limited resources or wealth, by using different methods. One method is to invest, in order to obtain payoffs with risk. It is a common belief that investments with a positive risk-return relationship (namely, high risk high return and vice versa) are dominant over those with a negative risk-return relationship (i.e., high risk low return and vice versa) in the human society; the belief has a notable impact on daily investing activities of investors. Here we investigate the risk-return relationship in a model complex adaptive system, in order to study the effect of both market efficiency and closeness that exist in the human society and play an important role in helping to establish traditional finance/economics theories. We conduct a series of computer-aided human experiments, and also perform agent-based simulations and theoretical analysis to confirm the experimental observations and reveal the underlying mechanism. We report that investments with a negative risk-return relationship have dominance over those with a positive risk-return relationship instead in such a complex adaptive systems. We formulate the dynamical process for the system's evolution, which helps to discover the different role of identical and heterogeneous preferences. This work might be valuable not only to complexity science, but also to finance and economics, to management and social science, and to physics.

## Introduction

One can see most of the social, ecological, and biological systems that contain a large number of interacting autonomous agents as complex adaptive systems (CASs), because the agents have adaptive capacities to the changing environment [1]. CAS dynamics have attracted much attention among physical scientists [2]–[6]. For survival and development, such agents in various kinds of CASs involving the human society must compete against or collaborate with each other for sharing limited resources or wealth, by utilizing different methods. One method is to invest, in order to obtain payoffs with risk. Accordingly, understanding the risk-return relationship (RRR) has not only an academic value but also a practical importance. So far this relationship has a two-fold character. On one hand, one considers investments as high risk high return and vice versa; the RRR is positive (risk-return tradeoff) [7], [8]. This is also an outcome of the traditional financial theory under the efficient market hypothesis. On the other hand, one also finds that some investments are high risk low return and vice versa; the RRR is negative (Bowman's paradox) [9], [10]. However, almost all investment products take “high risk high return” as a bright spot to attract investors, and neglect the possible existence of “high risk low return”. This actually results from a received belief that investments with a positive RRR are dominant over those with a negative RRR in the human society; the belief directs investors to operate daily investing activities including gambling [11]. Here we investigate the RRR by designing and investigating a model CAS which includes the following two crucial factors:

*Market efficiency.* The present system exhibits market efficiency at which it reaches a statistical equilibrium [5], [6]. We shall address more relevant details at the end of the next section.
*Closeness.* The system involves two conservations: one is the conservation of the population of investors (Conservation I), the other is the conservation of wealth (Conservation II). Regarding Conservation I/II, it means that we fix the total number/amount of the subjects/wealth in the system.

Clearly the two factors have real traces in the human society. Accordingly they have played an important role in helping to establish traditional finance/economics theories. The present designing system just allows us to investigate the joint effect of the two factors on the RRR.

## Results

### Human Experiment

On the basis of the CAS, we conduct a series of computer-aided human experiments. (These experiments are essentially online games, thus ethics approval was not necessary. In the mean time, we obtained verbal consent from all the subjects.) Details: there are two virtual rooms, Room 1 and Room 2 (represented by two buttons on the computer screen of the subjects), for subjects to invest in. The two rooms have volumes, 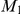 and 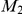, which may represent the arbitrage space for a certain investment in the real world. In the experiments, we recruited 24 students from Fudan University as subjects. The subjects acted as fund managers, who were responsible for implementing a fund's investing strategy and managing its trading activities. We told the subjects the requirement of total 30 rounds for each 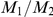, and offered every subject 1000 points (the amount of virtual money constructs the fund managed by the subject) as his/her initial wealth for each 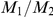. In an attempt to make the subjects maximize their pursuit of self-interest, we promised to pay the subjects Chinese Yuan according to the fixed exchange rate, 100∶1 (namely, one hundred points equal to one Chinese Yuan), at the end of the experiments, to offer every subject 30 Chinese Yuan as a bonus of attendance, and to give extra 50 Chinese Yuan to the subject who gets the highest score for one 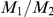. At the beginning of the 1st round of each 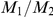, we told the 24 subjects the actual ratio of 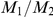, and asked each subject to decide his/her investing weight [signed as 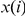 for Subject 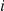]. Note the investing weight, 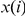, is the percentage of his/her investing wealth (investment capital) with respect to his/her total wealth, and it will keep fixed within the 30 rounds for a certain 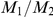. At every round, each subject can only independently invest in one of the two rooms. After all the subjects made their own decisions, with the help of the computer program, we immediately knew the total investments in each room (signed as 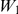 and 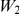 for Room 1 and Room 2, respectively) at this round. While keeping the total wealth conserved, we redistributed the total investment 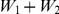 according to the following two rules:

1. We divided the total investment, 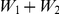, by the ratio of 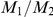, yielding 
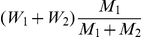
 and 
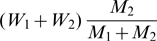
 as the payoff for Room 1 and Room 2, respectively.
2. We redistributed the payoff of Room 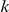 (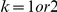) by the investment of the subjects. Namely, for each round, the payoff for Subject 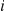 choosing Room 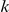 to invest in, 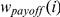, is determined by 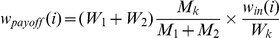, where 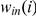 is the investing wealth of Subject 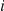, 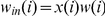. Here 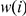 is the total wealth possessed by Subject 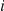 at the end of the previous round.

**Figure 1:**
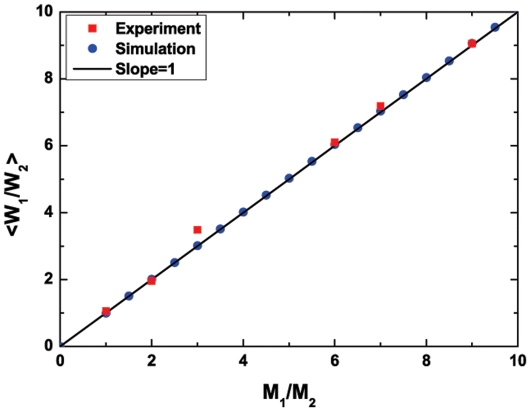
Averaged ratio, 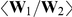
 **, versus** 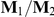 **for the human experiments with 24 subjects (red squares) and agent-based computer simulations with 1000 agents (blue dots).** Here “
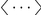
” denotes the average over the total 30 experimental rounds (experimental data of 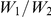 for each round are shown in Table 1) or over the 800 simulation rounds (the additional 200 rounds were performed at the beginning of the simulation for each M1/M2; during the 200 rounds, we train all of the strategies by scoring them whereas the wealth of each agent remains unchanged). All the experimental and simulation points lie in or beside the diagonal line (“slope = 1”), which is indicative of 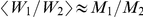. Parameters for the simulations: 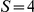 and 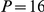.

Before the experiments, we told the subjects the above two rules for wealth re-allocation. After each round, every subject knows his/her payoff, 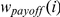. If there is 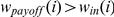, that is, Subject 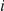 gets more than the amount he/she has invested, we consider Subject 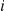 as a winner at this round. Equivalently, if 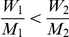, the subjects choosing Room 1 to invest in win at this round. Clearly, when 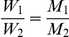, every subject obtains the payoff which equals to his/her investing wealth. Namely, the arbitrage opportunity has been used up. Accordingly, we define the 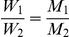 state as an equilibrium (or balanced) state [12]. This state may have some practical significance because global arbitrage opportunities for investing in the human society always tend to shrink or even disappear once known and used by more and more investors. As shown in Figure 1 (as well as Table 1), our experimental system can indeed achieve 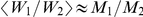 at which the system automatically produces the balanced allocation of investing wealth; this system thus reaches a statistical equilibrium. In other words, the “Invisible Hand” plays a full role [6], or alternatively the system exhibits market efficiency. That is, all subjects are pursuing self-interest and we run the present system under three conditions: with sufficient information (namely, the wealth change for each round has reflected the possible information), with free competition (i.e., no subjects dominate the system and there are zero transaction costs), and without externalities (the wealth change of a subject has reflected the influence of his/her behavior on the others).

**Table 1:** Experimental data of 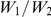
's for six 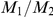
's within 30 rounds.

Round	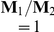	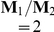	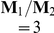	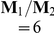	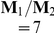	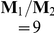
1	1.247723	1.143654	5.267782	2.98977	24.41429	2.146853
2	0.582237	0.702725	1.717598	11.02642	6.827457	4.860541
3	0.759914	1.897306	2.43237	10.32266	11.25343	11.30546
4	1.903253	1.240914	2.699907	2.97036	5.688661	9.681926
5	1.940527	1.564242	3.999681	3.977399	6.546176	5.249869
6	1.4852	4.711605	2.815152	6.900399	5.13295	6.16301
7	0.71966	2.087147	8.280381	2.991117	9.27272	7.25918
8	0.675138	1.692307	4.590899	3.35285	7.12301	8.996662
9	1.029128	2.73341	1.833477	4.363129	4.329496	7.133701
10	0.867554	2.095702	3.063358	7.273544	8.198398	14.26918
11	1.50125	1.305197	3.862686	18.23372	5.927536	5.500789
12	0.846259	2.292878	3.826587	8.50234	4.673143	5.141253
13	0.629585	1.992493	5.31337	4.613084	13.47519	34.4646
14	0.784858	2.462247	4.687499	19.73941	4.867279	3.889573
15	1.484235	1.807911	2.991726	3.40541	9.820732	7.442826
16	2.309969	1.544355	3.301258	4.864645	19.63957	15.74645
17	1.01251	2.078769	1.009523	8.219743	4.389477	11.55617
18	0.987891	2.624829	1.531467	2.935522	6.684373	8.712361
19	1.319123	2.25104	2.29988	3.813827	6.655679	6.623739
20	0.872338	2.045779	3.140856	5.690231	9.253236	7.973963
21	1.166773	2.006077	5.282071	5.889009	5.021116	5.825073
22	0.896165	1.419159	3.53215	6.137386	7.409623	8.32772
23	0.872224	2.141954	2.629218	11.09127	7.033376	15.57089
24	1.275063	1.990766	4.722947	5.989491	7.216511	10.87512
25	0.695696	2.151347	3.410795	7.790409	8.787551	4.759215
26	1.149307	2.150258	3.400615	8.213546	6.472158	13.14246
27	1.379602	1.621164	5.898509	5.078065	6.915495	7.992252
28	0.809361	1.62651	2.421057	3.698009	5.514453	11.76899
29	0.772988	1.670855	3.576442	7.848631	7.483899	16.27463
30	0.367173	2.010509	2.90843	11.10609	8.9996	4.854004

**Figure 2:**
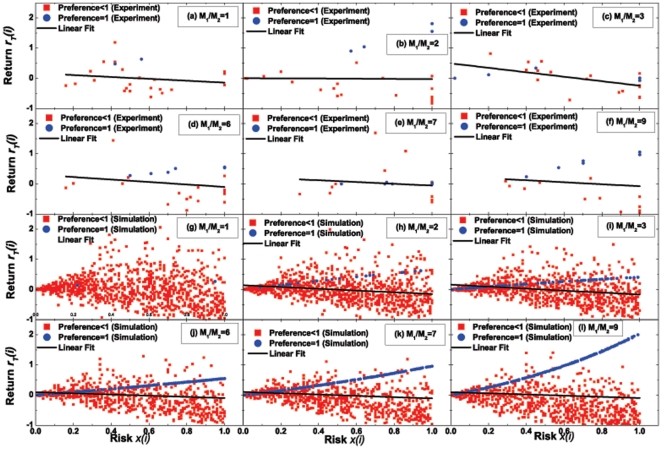
Relationship between the risk, 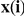
 **, and the return,** 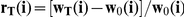 **, for (a)–(f) 24 subjects and (g)–(l) 1000 agents at various** 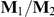 **'s.** (a)–(f) Data of the human experiments (total 30 rounds for each 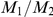); (g)–(l) Data of the agent-based computer simulations (total 800 rounds for each 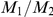, with additional 200 rounds performed at the beginning of the simulations; during the 200 rounds, we train all of the strategies by scoring them whereas the wealth of each agent remains unchanged). Here 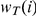 is Agent 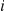
's wealth at the end of 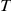 rounds (the total number of rounds, 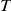, is 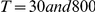 for the experiments and simulations, respectively), and 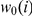 is Agent 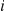
's initial wealth. All of the subjects or agents are divided into two groups with preference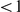 (red squares) and preference = 1 (blue dots). Here, the “preference” is given by 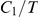, where 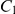 is the number of times for subjects or agents to choose Room 1 within the total 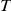 rounds. The values or distribution of the preferences of the subjects or agents can be found in Figs. 4 and 5. Here, “Linear Fit” denotes the line fitting the data in each panel using the least square method, which serves as a guide for the eye. (The fitting functions are listed in Table 2.) All of the lines are downward, which indicate a statistically negative relationship between risk and return. The present negative relationship just reflects the dominance of investments with a negative RRR in the whole system, in spite of a relatively small number of investments with a positive RRR. Other parameters: (g)–(l) 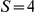 and 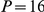.

If a subject (namely, a fund manager) chooses a larger investing weight, he/she will invest more virtual money in a room. According to the rules of our experiment, the room he/she chooses will then be more likely to be the losing one. Besides, the initial wealth is the same for every subject and he/she knows nothing but himself/herself. From this point of view, the larger investing weight he/she chooses, the higher risk (or uncertainty) he/she will take for the fund (i.e., the initial 1000 points). Therefore, throughout this work, we simply set the investing weight, 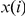, to equal the risk he/she is willing to take. Here we should remark that the present definition of risk appears to be different from that in finance theory. For the latter, one often defines risk according to variance. Nevertheless, the two kinds of risk are essentially the same because they both describe the uncertainty of funds and have a positive association with each other. On the other hand, we should mention that the risk we define for each subject does not change with the evolution of the time. This is a simplification which makes it possible to discuss the pure effect of a fixed value of “risk”. Nevertheless, if we choose to let the “risk” change with the time, for the same purpose, we may take an average of the “risk” over the full range of time. Figure 2(a)–(f) displays the risk-return relationship for the investments in the designing CAS. From statistical point of view, we find that investments with a negative RRR are dominant over those with a positive RRR in the whole system.

### An Agent-Based Model

Clearly the human experiments have some unavoidable limitations: specific time, specific avenue (a computer room of Fudan University), specific subjects (students from Fudan University), and the limited number of subjects. Now we are obliged to extend the experimental results [Figure 2(a)–(f)] beyond such limitations. For this purpose, we resort to an agent-based model [12]–[14].

Similar to the above experiments, we set two virtual rooms, Room 1 and Room 2 (with volume 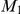 and 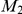, respectively), for 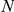 agents (fund managers) to invest in. Then, for each 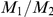, assign every agent 1000 points as his/her initial wealth and an investing weight, 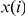, which is randomly picked up between 0 and 1 with a step size of 0.001. In order to avoid the crowding or overlapping of strategies of different agents [15]–[17], we design the decision-making process for each agent with four steps.

*Step 1:* set a positive integer, 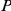, to represent the various situations for investing [5], [6].
*Step 2:* assign each agent 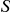 strategies according to 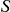 integers between 0 and 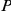, respectively. For example, if one of the 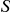 integers is 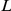, then the corresponding strategy of the agent is given by the ratio 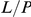 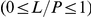, which represents the probability for the agent to choose Room 1 to invest in [5].
*Step 3:* for an agent, each strategy has its own score with an initial score, 0, and is added one score (or zero score) if the strategy predicts (or does not predict) the winning room correctly after each round.
*Step 4:* every agent chooses either Room 1 or Room 2 to invest in according to the prediction made by the strategy with the highest score.

In addition, both the payoff function and the rules for re-distributing investing wealth in Room 1 and Room 2 are set to be the same as those already mentioned in the section of Human Experiments.

### Comparison between Experimental and Simulation Results

As shown by Figure 1, our agent-based computer simulations also give 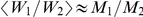, that is, the system under simulation also exhibits market efficiency. Further, according to the simulations, we achieve the same qualitative conclusion: investments with a negative RRR are statistically dominant over those with a positive RRR in the whole system; see Figure 2(g)–(l). Nevertheless, when we scrutinize Figure 2(j)–(l), we find that some particular data seem to be located on a smooth upward line. Then we plot these data in blue, and further find that they are just corresponding to all the agents with “preference = 1”. Encouraging by this finding, we blue all the data of “preference = 1” in the other 9 panels of Figure 2, and observe that a similar upward line also appears in the experimental results [see the blue dots in Figure 2(a)–(f); note the blue dots in Figure 2(c) and (e) are also, on average, in an upward line even though they appear to be not so evident].

For the upward lines themselves, they are clearly indicative of investments with a positive RRR. Hence, to distinctly understand our main conclusion about the dominance of investments with a negative RRR in the whole system, we have to overcome the puzzle, namely, the strange appearance of these upward lines (constructed by the blue dots in Figure 2). For convenience, we just need to answer Question 1: why do all the “preference = 1” data dots of Figure 2(g)–(l) exist in an upward line? To this end, the process to find the answer to Question 1 will also help to reveal the mechanism underlying the above main conclusion.

### Comparison among Experimental, Simulation, and Theoretical Results

**Figure 3:**
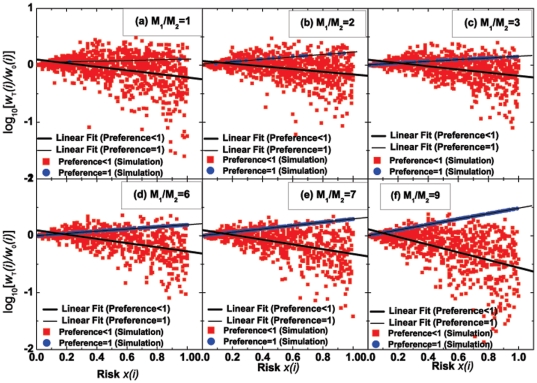
Same as **Figure 2(g)–(l)**, but showing the relationship between the risk, 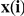
 **, and the relative wealth,** 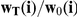 **, on a logarithmic scale.** “Linear Fit” corresponds to the line fitting the data of preference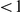 or preference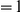 using the least square method, which serves as a guide for the eye. (The fitting functions are listed in Table 3.)

To answer Question 1, alternatively we attempt to study the relationship between risk and wealth; see Figure 3. In Figure 3, the “preference = 1” data dots appear to be arranged in an upward straight line, and the straight line exactly corresponds to the upward line constructed by the blue dots in Figure 2(g)–(l) due to the relationship between the wealth and return. So, Question 1 equivalently becomes Question 2: why do all the “preference = 1” data dots of Figure 3 exist in an upward straight line? To answer it, we start by considering Agent 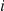 with investment weight, 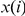. Then his/her return and wealth after 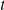 rounds are, respectively, 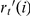 and 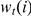. Here, the subscript 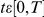. (Note 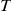 stands for the total number of simulation rounds, 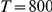.) Clearly, when 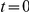, 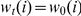, which just denotes the initial wealth of Agent 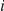. Then, we obtain the expression for 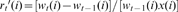. Accordingly, we have 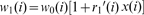 and 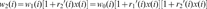, thus yielding 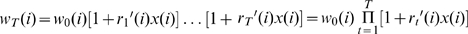. As a result, we obtain 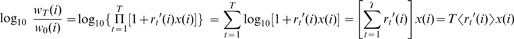. Here the third “ = ” holds due to 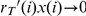 for the 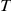 simulation rounds of our interest. In this equation, 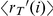 denotes the average return, namely, the value obtained by averaging 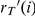 over the 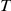 rounds, and 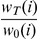 represents the relative wealth. Thus, the relationship between 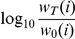 and 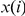 should be linear; the sign of the slope of the straight lines is only dependent on the average return, 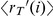. Because the agents with preference = 1 always enter Room 1 with 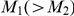, the average return, 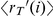, for them is not only positive but also the same. This is why all the blue points in Figure 3 lie on an upward straight line. However, for the other agents with preference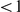 (Figure 3), they will change rooms from time to time, so their average return, 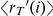, is different from one another. This is the reason why the red points do not form a straight line as the blue points do. From this point of view, the downward straight line we draw for the red points in Figure 3 is just a statistical analysis, showing a trend. They do not actually form a straight line. So far, our answer to Question 2 can simply be “because for the small number of agents with preference = 1, their average return, 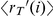, is not only positive but also the same”.

**Figure 4:**
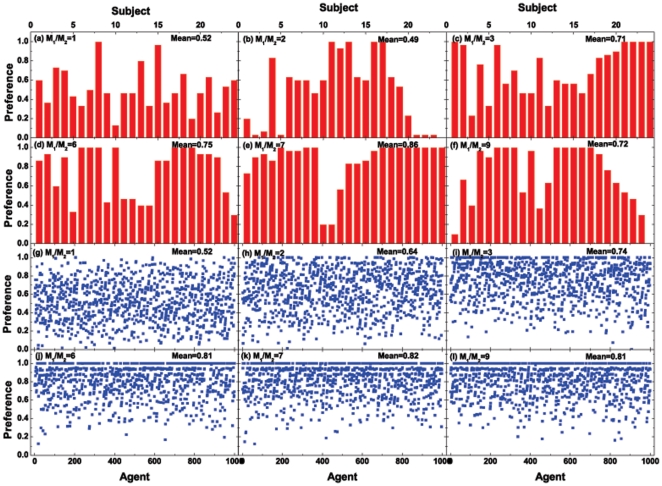
Preferences of (a)–(f) the 24 subjects in the human experiments (plotted in the bar graph) or (g)–(l) the 1000 agents in the agent-based computer simulations, for various 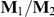
 **'s.** Here, “Mean” denotes the preference value averaged for (a)–(f) the 24 subjects or (g)–(l) 1000 agents. In (a)–(f), the present 24 subjects are ranked by their risk (namely, their investing weight) from low to high, within the range (a) [0.16, 1], (b) [0.01, 1], (c) [0.02, 1], (d) [0.16, 1], (e) [0.31, 1], and (f) [0.29, 1]; see Table 4 for details. Similarly, in (g)–(l), the 1000 agents are ranked by their risk from low to high, within the range (0, 1] assigned according to the code “(double)rand()
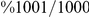
” in the C programming language. In (a)–(f), the ratio between the numbers of subjects with “preference = 1” and “preference
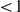
” are, respectively, (a) 2/22, (b) 4/20, (c) 5/19, (d) 7/17, (e) 11/13, and (f) 8/16. In (g)–(l), the ratio between the numbers of agents with “preference = 1” and “preference
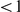
” are, respectively, (g) 2/998, (h) 23/977, (i) 94/906, (j) 233/767, (k) 200/800, and (l) 220/780.

**Figure 5:**
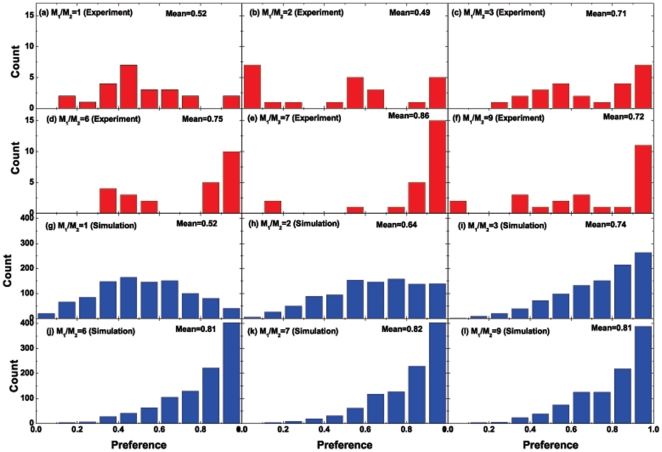
Same as **Figure 4**, but showing the distribution of preferences.

According to the above theoretical analysis, we can now understand that the statistical dominance of investments with a negative RRR in the whole system results from the distribution of subjects'/agents' preferences: the heterogeneous preferences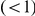 owned by a large number of subjects/agents together with the identical preferences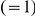 possessed by a small number of subjects/agents. Details about the actual values for the preferences can be found in Figs. 4–5. Figs. 4–5 also show the environmental adaptability of subjects or agents.

## Discussion

On the basis of the designed CAS (complex adaptive system), we have revisited the relationship between risk and return under the influence of market efficiency and closeness by conducting human experiments, agent-based simulations, and theoretical analysis. We have reported that investments with a negative RRR (risk-return relationship) have dominance over those with a positive RRR in this CAS. We have also revealed the underlying mechanism related to the distribution of preferences. Our results obtained for the overall system do not depend on the evolutionary time, 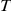, as long as 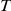 is large enough. On the other hand, the experimental data for each 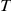 have been listed in Table 1. Clearly, the results for each 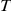 can change accordingly. In fact, such changes echo with those fluctuations or volatilities yielding arbitrage opportunities for investors in the real human society.

This work should be valuable not only to complexity science, but also to finance and economics, to management and social science, and to physics. In finance and economics, it may remind investors about their daily investing activities. In management and social science, our work is of value on clarifying the relationship between risk and return under some conditions. In physics, the present work reveals a new macroscopic equilibrium state in such a CAS.

**Table 2:** Linear fitting functions for Figure 2.

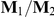	For the experimental data [Fig. 2(a)–(f)]	For the simulation data [Fig. 2(g)–(l)]
1	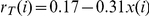 [Fig. 2(a)]	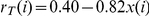 [Fig. 2(g)]
2	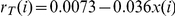 [Fig. 2(b)]	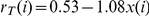 [Fig. 2(h)]
3	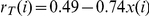 [Fig. 2(c)]	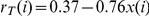 [Fig. 2(i)]
6	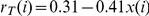 [Fig. 2(d)]	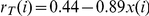 [Fig. 2(j)]
7	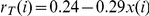 [Fig. 2(e)]	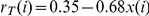 [Fig. 2(k)]
9	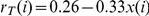 [Fig. 2(f)]	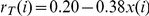 [Fig. 2(l)]

**Table 3:** Linear fitting functions for Figure 3.

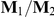	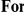 “ 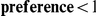 ”	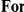 “ 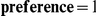 ”
1	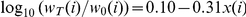 [Fig. 3(a)]	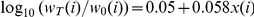 [Fig. 3(a)]
2	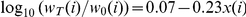 [Fig. 3(b)]	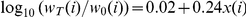 [Fig. 3(b)]
3	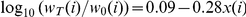 [Fig. 3(c)]	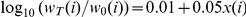 [Fig. 3(c)]
6	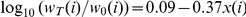 [Fig. 3(d)]	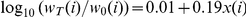 [Fig. 3(d)]
7	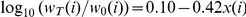 [Fig. 3(e)]	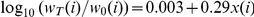 [Fig. 3(e)]
9	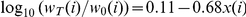 [Fig. 3(f)]	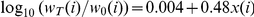 [Fig. 3(f)]

**Table 4:** Values for the risk (namely, investing weight) of the 24 subjects for six 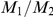
's in the human experiments.

Subject	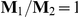 [Fig. 4(a)]	2[Fig. 4(b)]	3[Fig. 4(c)]	6[Fig. 4(d)]	7[Fig. 4(e)]	9[Fig. 4(f)]
1	0.16	0.01	0.02	0.16	0.31	0.29
2	0.21	0.02	0.2	0.2	0.46	0.31
3	0.29	0.11	0.21	0.41	0.47	0.39
4	0.31	0.2	0.4	0.46	0.49	0.4
5	0.36	0.26	0.41	0.49	0.52	0.47
6	0.42	0.41	0.45	0.5	0.7	0.57
7	0.42	0.48	0.46	0.61	0.75	0.7
8	0.42	0.5	0.46	0.7	0.75	0.7
9	0.46	0.5	0.48	0.7	0.79	0.71
10	0.47	0.52	0.5	0.74	0.86	0.74
11	0.48	0.57	0.63	0.76	1	0.79
12	0.5	0.6	0.72	0.8	1	0.9
13	0.5	0.64	0.74	0.8	1	1
14	0.55	0.74	0.89	0.82	1	1
15	0.56	0.81	0.91	0.86	1	1
16	0.61	1	1	0.86	1	1
17	0.61	1	1	1	1	1
18	0.63	1	1	1	1	1
19	0.66	1	1	1	1	1
20	0.67	1	1	1	1	1
21	0.72	1	1	1	1	1
22	1	1	1	1	1	1
23	1	1	1	1	1	1
24	1	1	1	1	1	1

We ranked the 24 subjects by their risk from low to high, as already used in Figure 4(a)–(f).
